# Seemingly trivial secondary factors may determine microbial competition: a cautionary tale on the impact of iron supplementation through corrosion

**DOI:** 10.1093/femsec/fiab002

**Published:** 2021-01-11

**Authors:** Gerben R Stouten, Kelly Hamers, Rinke J van Tatenhove-Pel, Eline van der Knaap, Robbert Kleerebezem

**Affiliations:** Department of Biotechnology, Delft University of Technology, van der Maasweg 9, 2629 HZ, Delft, The Netherlands; Department of Biotechnology, Delft University of Technology, van der Maasweg 9, 2629 HZ, Delft, The Netherlands; Department of Biotechnology, Delft University of Technology, van der Maasweg 9, 2629 HZ, Delft, The Netherlands; Amsterdam Institute for Molecules, Medicines and Systems, Vrije Universiteit Amsterdam, De Boelelaan 1108, 1081 HZ, Amsterdam, The Netherlands; Department of Biotechnology, Delft University of Technology, van der Maasweg 9, 2629 HZ, Delft, The Netherlands; Department of Biotechnology, Delft University of Technology, van der Maasweg 9, 2629 HZ, Delft, The Netherlands

**Keywords:** enrichments, PHA, bioavailability iron, microbial competition, corrosion

## Abstract

Microbial community engineering aims for enrichment of a specific microbial trait by imposing specific cultivation conditions. This work demonstrates that things may be more complicated than typically presumed and that microbial competition can be affected by seemingly insignificant variables, like in this case the type of acid used for pH control. Aerobic bioreactors pulse fed with acetate operated with hydrochloric acid resulted in the enrichment of *Plasticicumulans acidivorans*, and changing the pH controlling agent to sulfuric acid shifted the community towards *Zoogloea sp*. Further research demonstrated that the change in community structure was not directly caused by the change in acid used for pH control, but resulted from the difference in corrosive strength of both acids and the related iron leaching from the bioreactor piping. Neither system was iron deficient, suggesting that the biological availability of iron is affected by the leaching process. Our results demonstrate that microbial competition and process development can be affected dramatically by secondary factors related to nutrient supply and bioavailability, and is way more complex than generally assumed in a single carbon substrate limited process.

## Teaser

Apparently insignificant cultivation differences can result in major changes in microbial community structure and functioning.

## INTRODUCTION

Microbial community engineering (MCE) utilizes ecological selection principles to enrich microbial communities with specific functional properties, e.g. the production of chemicals and bioenergy (Kleerebezem and van Loosdrecht 2007). MCE finds its roots in the work of Baas Becking: ‘Everything is everywhere, but the environment selects’ (Baas-Becking 1934). By using selective environments, we aim to enrich and maintain microbial communities with desired functionalities under non-axenic, i.e. open, conditions. MCE can contribute to the circular economy and valorize non-sterilized mixed substrate streams, thereby unlocking the tremendous carbon and energy resources originating from heterogeneous feedstocks currently regarded as waste streams.

In general, laboratory enrichment studies are designed with the idea in mind that one substrate is present in the influent in a rate determining concentration. Typically, in selective conditions favoring heterotrophic growth, the limiting substrate is the carbon and energy source. All other essential growth nutrients are supplied in excess with the objective to characterize the process as a function of a single substrate limitation. In this way, microbial competition is assumed to be investigated as a function of a single variable, and conclusions can be drawn in terms of the dependency of system development on this variable. For example, the competition in chemostat enrichment experiments generally is assumed to be determined by the affinity for one limiting substrate. To which extent this assumption holds true is rarely verified due to the large number of medium constituents that would need to be tested. Nevertheless, it remains largely unclear if microbial ecosystem development depends on the concentration and bioavailability of secondary substrates such as trace elements.

In this work, we describe our analysis of an unanticipated secondary limitation encountered in experiments aiming for enrichment of a polyhydroxyalkanoates (PHA) producing microbial community. PHA is a polymer with chemical properties that make it an interesting bioplastic that is fully biodegradable (Chen 2009; Tamis *et al*. 2018). Enrichment of PHA producing microorganisms can be established by aerobic cultivation in alternating presence and absence of the carbon substrate. Over the past 10 years, this strategy was shown repeatedly to enable the effective enrichment of the superior PHA-producer *Plasticicumulans acidivorans* from sewage sludge, and it has been elemental to mixed culture PHA research (Johnson, Kleerebezem and van Loosdrecht 2009; Jiang *et al*. 2011; Tamis *et al*. 2014; Marang, van Loosdrecht, and Kleerebezem 2016; Stouten *et al*. 2019). Typically, the enrichment of *P. acidivorans* from activated sludge can be established within 30 generations (Stouten *et al*. 2019). In a new attempt to enrich for *P. acidivorans* we operated a Sequencing Batch Bioreactor (SBR) for more than 60 generations. Although conditions known to enable effective enrichment of *P. acidivorans* were applied, no enrichment of *P. acidivorans* was established and the functional performance in terms of substrate conversion rates was markedly different. The sole operational difference with previous systems was the choice of acid used for pH control; Previous enrichments were conducted with HCl, while in this enrichment H_2_SO_4_ was used. Based on this observation we decided to investigate in more detail the role of the type of acid used for pH control in the process, with the objective to identify the secondary factors that determine enrichment and functional process development.

## MATERIALS AND METHODS

### Sequencing batch bioreactor operation

Two microbial enrichments using acetate as carbon source and electron donor, and oxygen as electron acceptor, were established in sequencing batch bioreactors (SBR) under a feast-famine regime at 30°C, pH 7, cycle length 12 h and exchange ratio of 50%. This operation results in a solid retention time (SRT) of approximately 24 h. The reactor set-up and operation are described in detail by Johnson *et al*. (2009). Bioreactors were inoculated with activated sludge from a sewage treatment facility (Harnaschpolder, Delft, the Netherlands) and were operated at a working volume of 1.4 L (Applikon the Netherlands), where each cycle started with 5 min of discharging 700 mL of mixed reactor content, feeding 640 mL of demineralized water, 20 mL of nutrient medium (250 mM NH_4_Cl, 25 mM KH_2_PO_4_, 6.25 mM MgSO_4_·7H_2_O, 7.50 mM KCl, 50 mL L^−1^ trace elements solution (Vishniac and Santer 1957)), and after one hour a 20 mL carbon pulse (1 M sodium acetate) is dosed in 1 minute. Throughout the cycle approximately 20 mL of acid and base is dosed for pH control, resulting in the final volume of 1.4 L. The stability of the cultures in terms of conversion rates and microbial community composition was monitored by regular sampling and via online monitoring of off-gas composition and acid and base (0.5 M NaOH) dosage. The two main enrichment bioreactor systems were operated in an identical fashion with the exception of acid source for pH control. ${\textit{SBR}_{HCl}}$ and ${\textit{SBR}_{{H_2}S{O_4}}}$ were operated with 1 M HCl, and 0.5 M H_2_SO_4_ as acid dosing agent, respectively.

### Shift experiments

During the effluent phase of each operational cycle, half of the reactor content is discharged. This broth was used as the starting culture (inoculum) for shift experiments in three separate bioreactors without disturbing the enrichments. Transfer of half of the biomass to a new bioreactor was verified to result in highly comparable performance in both bioreactors. Shift experiments are operated as identical replicates of the main enrichments, except for a single change in medium composition. Eight types of shift experiments were performed where the acid dosing agent was changed or different salts were added to the medium (Table 1). Shift experiments were operated for at least 20 operational cycles, after the initial change in medium composition.

**Figure 2.**
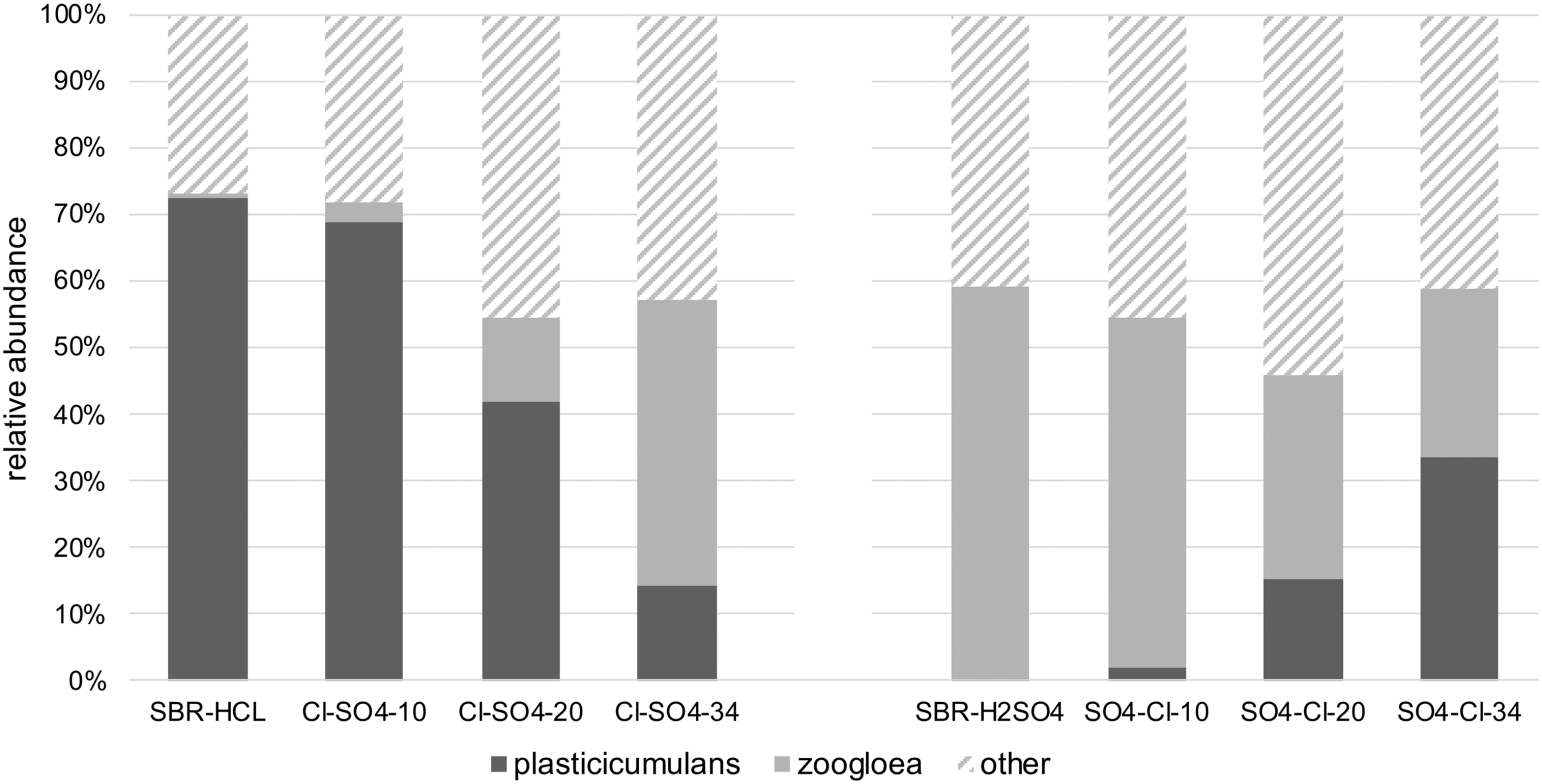
Relative abundance of 16S rRNA genes of *Plasticicumulans* and *Zoogloea* genera at several timepoints. ${{{\mathbf{SBR}}}_{{\bm{HCl}}}}$ and ${{{\mathbf{SBR}}}_{{{\bm{H}}_2}{\bm{S}}{{\bm{O}}_4}}}$ represent the relative community structure at the end of the enrichment period. The three following samples indicate samples at 10, 20 and 34 cycles after switching the relative acids for pH control. For graphing purposes, all other genera are combined under *other*, a more detailed overview of the relative community structure is given in the Supplementary Materials.

**Table 1.** Operational changes in acid, salt and metal added during shift experiments.

Shift#	Origin	Acid	Salt
1^a^	${\textit{SBR}_{HCl}}$	H_2_SO_4_	
2^a^	${\textit{SBR}_{{H_2}S{O_4}}}$	HCl	
3^b^	${\textit{SBR}_{HCl}}$	HCl	Na_2_SO_4_
4^b^	${\textit{SBR}_{{H_2}S{O_4}}}$	H_2_SO_4_	NaCl
5^b^	${\textit{SBR}_{HCl}}$	H_2_SO_4_	NaCl
6^c^	${\textit{SBR}_{HCl}}$	H_2_SO_4_	Ni(II)SO_4_.H_2_O
7^c^	${\textit{SBR}_{HCl}}$	H_2_SO_4_	Cr(III)K(SO_4_)_2_.2H_2_O
8^c^	${\textit{SBR}_{HCl}}$	H_2_SO_4_	Fe(II)Cl_2_

a Acid shift experiment where HCl and H_2_SO_4_ are interchanged.

b Anion change experiment with chloride and sulfate.

c Metal leaching experiment with artificial leaching substrates.

In some of the shift experiments specific trace metals were dosed throughout the cycle from three stock solutions: 1.5 mM CrK(SO_4_)_2_.2H_2_O, 1 mM NiSO_4_.H_2_O, and 5 mM Fe(II)Cl_2_. Titration was conducted through continuous dosing at 0.42 mL h^−1^ (5 mL per cycle). If no response was observed within 5 cycles, the flow rate was gradually increased to 1.67 mL h^−1^.

### Analytical procedures

Samples from the reactor for analysis of acetate, ammonium, iron, chromium, and nickel were immediately centrifuged (2 min. 10.000g) and filtered after sampling (0.45 μm pore size poly-vinylidene difluoride membrane, Merck Millipore, Carrigtohill, Ireland). The acetate concentration was measured by HPLC (BioRad Aminex HPX-87H column, Waters 2489 UV/RI detector, 1.5mM H_3_PO_4_ mobile phase with a flow rate of 0.6 mL min^−1^ and a temperature of 60°C). Ammonium, iron (II/III), chromium and nickel concentrations were determined spectrophotometrically using cuvette test kits (Hach Lange, Düsseldorf, Germany). The chromium, nickel and iron concentrations were determined after digestion at a pH of 2, for 60 minutes at 100°C. PHA content was measured as described by Johnson *et al*. 2009. Concentrations of N_2_, O_2_, Argon and CO_2_ in the off-gas of the reactor were measured online using mass spectrometry (Prima BT, Thermo Scientific).

### Calculations

Characterization of biological functioning of the microbial community is performed by reconstructing the oxygen uptake rate and carbon evolution rate profiles in each cycle based on online gas measurements as described in Stouten, Douwenga, *et al*. (2020). Online characterization allows discerning the extent of growth and PHA production in a cycle by correlating an increase in respiration activity with growth, and respiration in absence of extracellular substrate with metabolism of PHA. For all calculations the carbon mass balance and electron balance close within 95%.

### Microbial community structure and microscopy

The taxa-based microbial community composition of the enriched cultures was determined by amplicon sequencing of the 16S rRNA gene following the procedure described in (Stouten *et al*. 2019) and the sequences are available at NCBI under BioProject accession number [PRJNA577272].

Microscopy pictures were taken using a Leica DM500B light microscope (Leica Microsystems, Wetzlar, Germany) equipped with fluorescence filtercube A. 1 μl BODIPY 505/515 (Invitrogen D3921, Life Technologies, Grand Island, USA) in DMSO (1 mg ml^−1^) was used to stain PHA inclusion bodies in bacterial cells in 1 mL bioreactor sample.

## RESULTS

### Preliminary results giving rise to the research described in this paper

**Figure 1.**
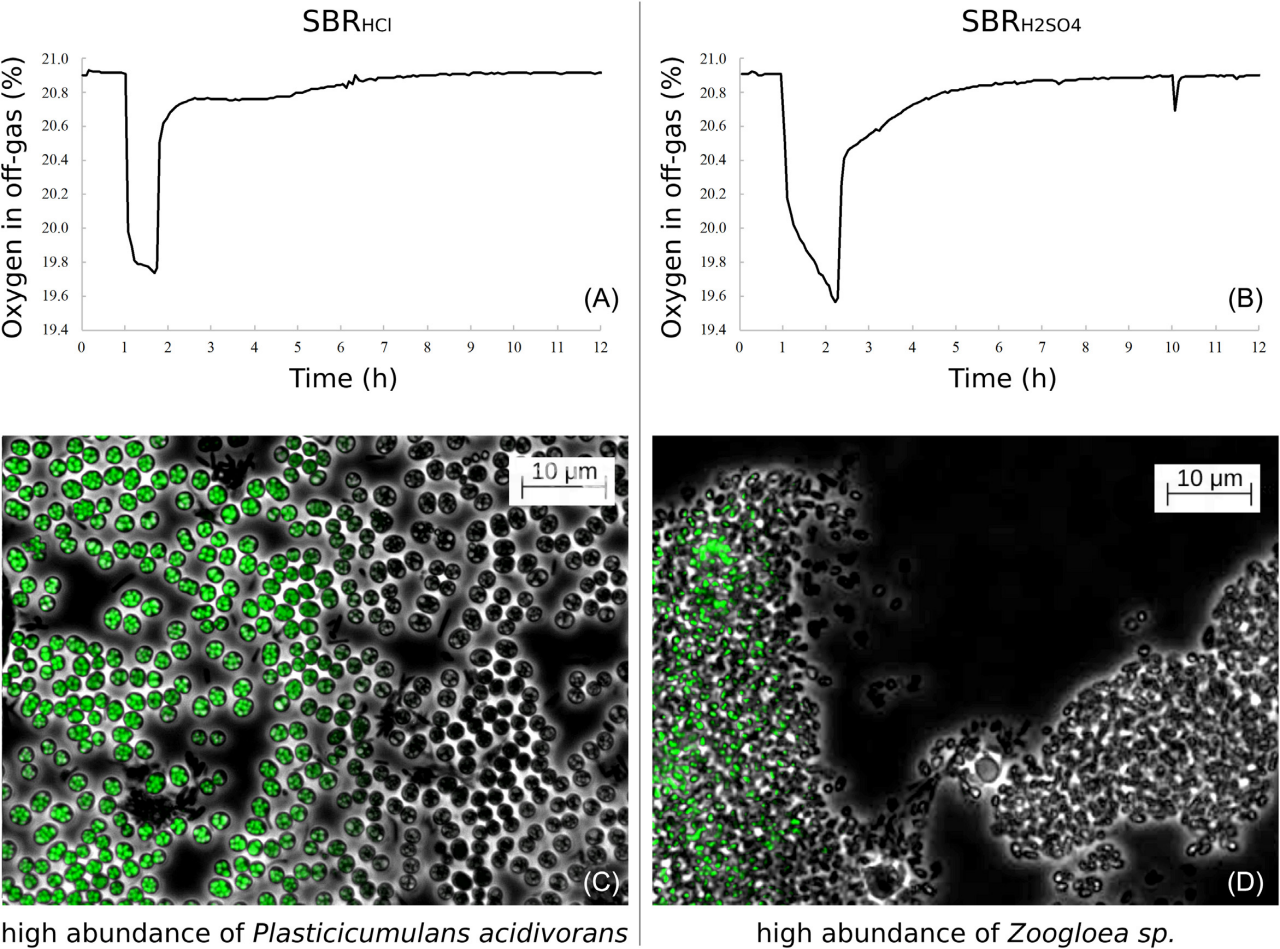
Respiration profiles and microbial morphological differences between the enrichment of ${{{\mathbf{SBR}}}_{{\bm{HCl}}}}$ (left) and ${{{\mathbf{SBR}}}_{{{\bm{H}}_2}{\bm{S}}{{\bm{O}}_4}}}$ (right). The oxygen off-gas profiles during steady state cycles are shown at the top **(A, B)**. Microscopic images of the microbial cultures at the end of the feast phase in dark field and fluorescent BODIPY 505/515 PHA staining are shown half overlapping to clearly show the PHA granules (green) and the cellular morphology differences of the enriched microbial cultures **(C, D)**.

In an attempt to establish a PHA producing microbial community dominated by *P. acidivorans*, an SBR was inoculated with activated sludge and operated at 30°C, pH 7, and a cycle length of 12 h. This strategy had repeatedly been demonstrated to enable *P. acidivorans* enrichment. However, in this case *P. acidivorans* was not observed as dominant community member and the functional behavior of the community as reflected in the dissolved oxygen patterns throughout the cycle was markedly different from *P. acidivorans* dominated enrichments, despite prolonged enrichment (Fig. 1).

The only operational difference we could identify from previous enrichment efforts was the acid used for pH control: H_2_SO_4_ was used in this study to minimize corrosion of the bioreactor-inlet, whereas we used HCl in the past. To investigate if the used acid determined the community structure and function, two new enrichments were started with HCl and H_2_SO_4_ as pH controlling agent.

### Differences between steady state enrichments with HCl and H_2_SO_4_ as acid used for pH control

Two microbial communities were enriched from activated sludge using acetate as carbon source and electron donor, and oxygen as electron acceptor. The sequencing batch reactors (SBR) were operated for 90 days resulting in the development of a feast-famine regime. Operationally, the only difference between system ${\textit{SBR}_{HCl}}$ and ${\textit{SBR}_{{H_2}S{O_4}}}$ was the choice of acid for pH control, hydrochloric acid and sulfuric acid respectively. Both enrichments achieved a pseudo steady state characterized by constant conversion rates and yields (within 5% variation) (Table 2). Sequencing of the 16S rRNA genes of the microbial community demonstrated enrichment of stable communities dominated by *Plasticicumulans acidivorans* in ${\textit{SBR}_{HCl}}$ and *Zoogloea sp*. in ${\textit{SBR}_{{H_2}S{O_4}}}$ (Fig. 2). Morphological differences between the two genera were clearly observed through microscopy (Fig. 1).

**Table 2.** Overview of the characteristic differences between the enrichment of ${{{\mathbf{SBR}}}_{{\bm{HCl}}}}$ and ${{{\mathbf{SBR}}}_{{{\bm{H}}_2}{\bm{S}}{{\bm{O}}_4}}}$. Standard deviations of eight cycles in parenthesis.

	unit	${\textit{SBR}_{HCl}}$	${\textit{SBR}_{{H_2}S{O_4}}}$
Dominant species	-	*P. acidivorans*	*Zoogloea sp*.
Length of feast phase	min.	40 (4)	80 (6)
Respiration ratio^a^	%	90 (3)	60 (5)
Ammonium consumed in feast^b^	%	5 (1)	38 (6)
Oxygen consumption in feast^b^	%	47 (4)	60 (5)
PHA end feast^c^	%	52 (3)	30 (7)
Maximum PHA accumulation^c^	%	89	78

a Ratio of oxygen transfer rate at the start of the feast-phase to the end of the feast-phase .

b Percentage of the total ammonium or oxygen consumed throughout the cycle.

c Weight percentage (g g_DW_^-1^) of PHA of volatile suspended solids (VSS) (Jiang *et al*. 2011).

Functionally, ${\textit{SBR}_{HCl}}$ showed the typical hoarding strategy (Stouten *et al*. 2019). During the feast phase, carbon was converted into PHA within 40 minutes, and negligible growth occurred during substrate uptake as reflected in negligible ammonium uptake and a constant respiration rate (Fig. 1 and Table 2). New catalytic biomass was produced in the famine phase, where storage polymers were converted into new biomass in the absence of an extracellular carbon source.

${\textit{SBR}_{{H_2}S{O_4}}}$ showed a mixed behavior, where storage polymers and catalytic biomass were produced during the 80 minutes feast phase, significant ammonium was taken up and the respiration rate increased (Fig. 1 and Table 2). In the famine phase the stored polymers were converted into catalytic biomass.

### Inverting the acids used for pH control

**Figure 3.**
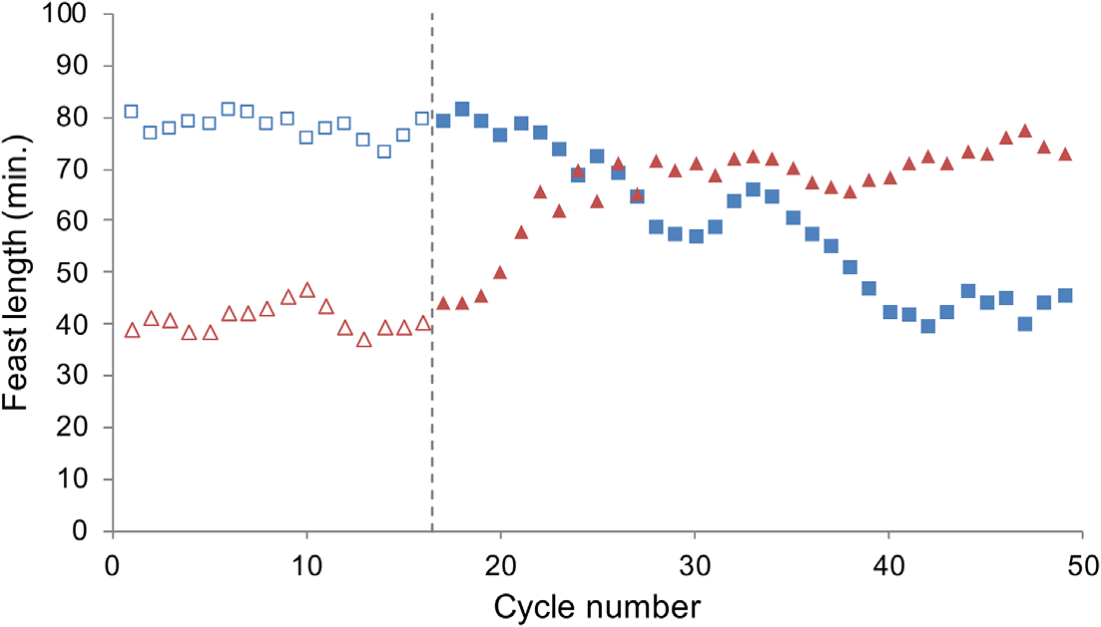
Change in feast length of ${{{\mathbf{SBR}}}_{{\bm{HCl}}}}$ (red triangles) and ${{{\mathbf{SBR}}}_{{{\bm{H}}_2}{\bm{S}}{{\bm{O}}_4}}}$ (blue squares) when switching their respective acids for pH control. Open figures represent the steady state feast length of fifteen cycles prior to the acid shift, the dashed line at cycle 16 indicates the moment where the acids were changed.

Biomass from ${\textit{SBR}_{HCl}}$ and ${\textit{SBR}_{{H_2}S{O_4}}}$ was used as inoculum for two additional bioreactors switching HCl with H_2_SO_4_ and vice versa. The microbial community structure in both systems shifted, demonstrating that the acid used for pH control determined the microbial community structure established, in a comparable timeframe (Fig. 2). Also, a shift in functionality of both systems was observed in response to the change in acid used for pH control (Table 2), but in a different timeframe (Fig. 3). ${\textit{SBR}_{{H_2}S{O_4} \to HCl}}$ showed a gradual shift in functionality as reflected by the decrease in the length of the feast phase, which was accompanied by a corresponding increase in relative abundance of *P. acidivorans*. ${\textit{SBR}_{HCl \to {H_2}S{O_4}}}$showed a rapid increase in length of the feast phase (within five cycles comparable to ${\textit{SBR}_{{H_2}S{O_4}}}$), but the microbial community remained dominated by *P. acidivorans* for at least 20 cycles after the switch from HCl to H_2_SO_4_. After 20 cycles, microscopy showed a decrease in community dominance of *P. acidivorans*, and after 34 cycles a shift towards a *Zoogloea sp*. dominated culture had occurred as supported by 16S rRNA gene sequencing (Fig. 2).

**Figure 4.**
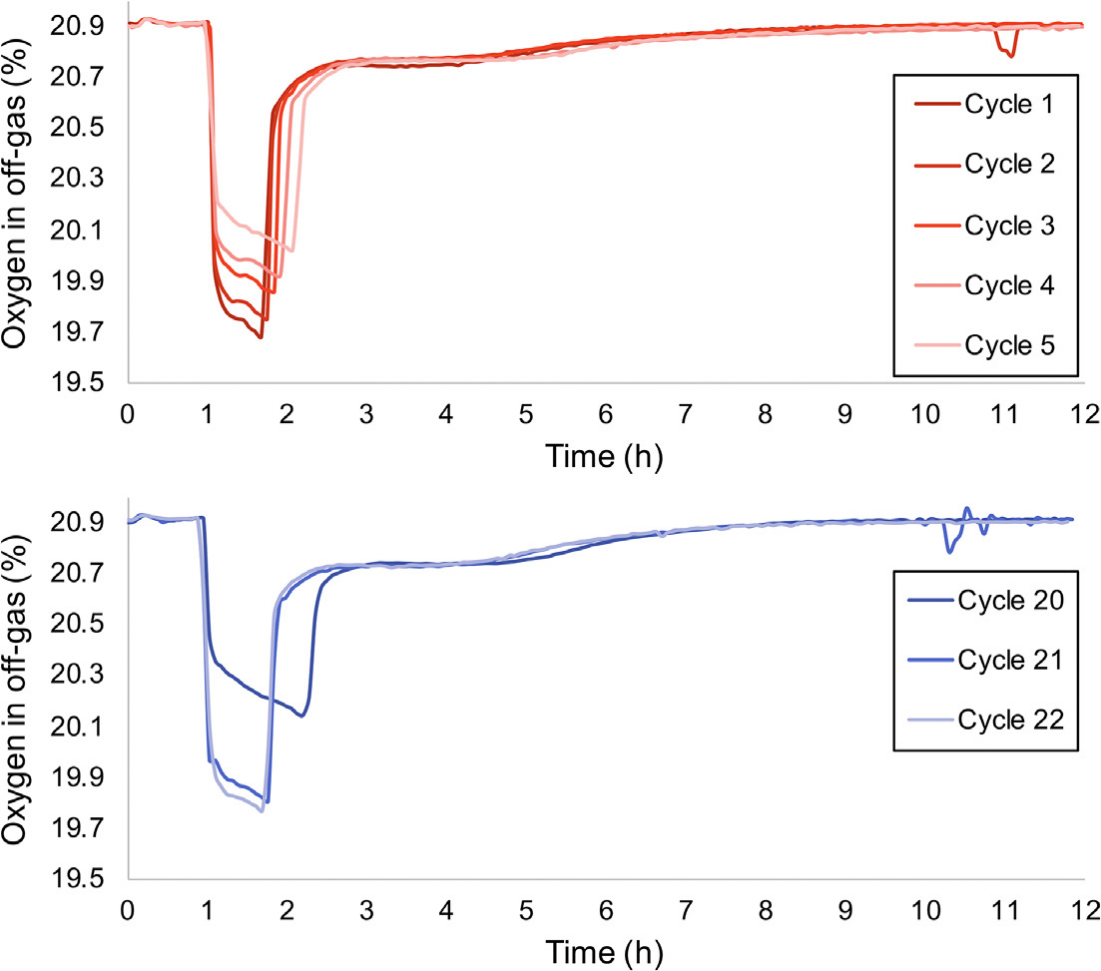
Overlay of oxygen off-gas profiles of ${{{\mathbf{SBR}}}_{{\bm{HCl}}}}$ after switching from HCl to H_2_SO_4_ for acid control (top—red) and switching back to HCl (bottom—blue) after 20 cycles of operation with H_2_SO_4_. The color gradient from dark to light indicates sequential cycles. In the HCl controlled system the feast length initially takes around 40 min, but increases to 80 min over 5 cycles when switching to H_2_SO_4_. The longer feast length, lower initial respiration rate and increasing respiration activity during the feast phase indicate a functional shift from solely PHA production to mixed PHA production and catalytic biomass production as detailed in Fig. 1.

Switching within 20 cycles the acid agent in ${\textit{SBR}_{HCl \to {H_2}S{O_4}}}$ back to HCl reduced the length of the feast phase to the original values of ${\textit{SBR}_{HCl}}$ within two operational cycles (Fig. 4). This nearly instantaneous change in functionality occurred even though a shift in the microbial community was only observed after 10 generations. This response was achieved repeatedly with biomass from ${\textit{SBR}_{HCl}}$ when the acid used for pH control was changed to H_2_SO_4_. We define this period as the transition state; a state in which the microbial community of ${\textit{SBR}_{HCl}}$ was not perceived to have changed, but the functionality did change as a result of the switch in acid agent.

### Chloride and sulfate ions

Switching the acid agent for pH control between HCl and H_2_SO_4_ results in a change in anions concentration in the reactor broth. Additionally, the divalent sulfate ions result in a slightly lower ionic strength compared to monovalent chlorine. To investigate the impact of the dominant anions in the system, sulfate and chloride sodium-salts were added to the medium of ${\textit{SBR}_{HCl}}$ and ${\textit{SBR}_{{H_2}S{O_4}}}$ respectively. By these means the eventual salt composition and concentration were identical in both systems. The process performance and microbial community structure in ${\textit{SBR}_{HCl}}$ was not affected by supplementing the medium with Na_2_SO_4_ during 50 operational cycles (data not shown). The enrichment performance of the ${\textit{SBR}_{{H_2}S{O_4}}}$ changed slightly with the addition of 20 mM NaCl as reflected in a feast length reduction from 80 to 75 min after 15 cycles. Overall, only a minor functional effect of the salt composition and concentration was observed. From these experiments it was concluded that the dominant anion in the process could not explain the differences in microbial community structure and operational properties between ${\textit{SBR}_{HCl}}$ and ${\textit{SBR}_{{H_2}S{O_4}}}$.

### Abiotic metal pitting corrosion

The reasoning for switching from HCl to H_2_SO_4_ during cultivation was to reduce the corrosive effect of HCl on the stainless-steel inlet feed triplet of the bioreactors (SAE 316L stainless steel, Applikon, Delft, The Netherlands) (Kovach 2002). The triplets needed to be replaced every 6–8 months when operated with 1 M HCl due to corrosion induced leakage (Fig. S2: left, Supporting Information). A secondary effect of the corrosion is the leaching of trace metals in the fermentation broth. To investigate how much trace metals are added over time, small parts from a leaking inlet triplet were cut off using electrical pliers and immersed in the acids that were used for pH control, 1 M HCl and 0.5 M H_2_SO_4_. Over a period of 30 days an obvious change due to corrosion of steel was observed in the HCl bottle: the clear acid solution became blue and the metal part turned black. The liquid and metal in the bottle with H_2_SO_4_ remained visually unaffected (Fig. S2: right, Supporting Information).

From the chemical composition of 316L stainless steel (Supplementary Table 1), iron, chromium, and nickel are present in significant quantities (>10 wt%) and are therefore the most likely candidates to have an impact on the microbial cultures (Oberg and Jones 1916). The concentrations in the HCl bottle after 30 days were determined to be 4.6 mM Fe, 1.4 mM Cr and 0.8 mM Ni. Concentrations in the H_2_SO_4_ bottle were below the detection limit (chromium <0.5 μM,  nickel <1.6 μM),  except for iron which was determined to be 50 μM. Approximate calculations suggest that operation of the SBR with 1M HCl can result in a leaking inlet within 500–1000 operational cycles (calculation described in Table S3, Supporting Information), which agrees with the observed failure rate of these inlets. The additionally titrated iron is within the same order of magnitude as iron dosed in the nutrient medium, chromium and nickel are not present in the nutrient medium.

### Titration of nickel and chromium

**Figure 5.**
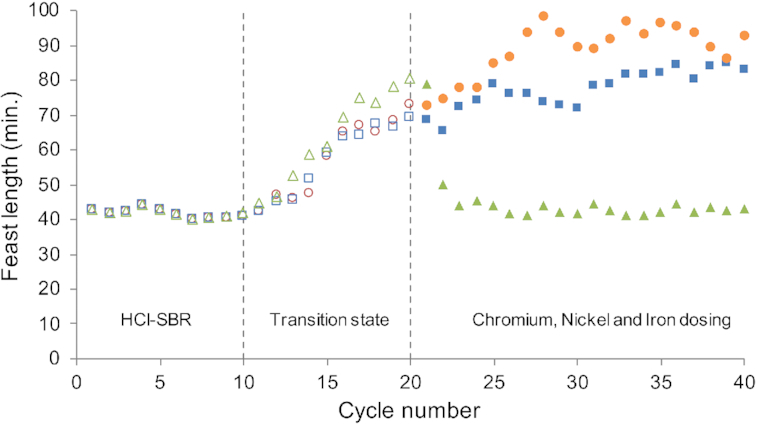
Feast length of *P. acidivorans* dominated enrichments, cultivated with continuous dosage of chromium (orange circles), nickel (blue squares) or iron (green triangles). Biomass from a stable enrichment in ${{{\mathbf{SBR}}}_{{\bm{HCl}}}}$ (cycles 1–10) was cultivated for ten cycles by replacing HCl with H_2_SO_4_ as acid source (cycles 10–20). From cycle 20 on, the bioreactors were supplemented with increasing amounts of chromium, nickel or iron, up to final concentrations of 40, 30 and 35 μM, respectively.

Chromium and nickel are not included in the medium and may therefore affect the microbial community when leached in the fermentation broth due to pH control with HCl. In two transition experiments, biomass from ${\textit{SBR}_{HCl}}$was transferred to a separate bioreactor and HCl was replaced with H_2_SO_4_ as pH controlling agent. In both bioreactors, the functional behavior changed towards the transition state within 10 cycles. Starting 10 cycles after the acid switch, increasing amounts of chromium or nickel were continuously titrated to the bioreactor to final concentrations of 40 μM and 30 μM respectively. These final concentrations were estimated from the leaching experiments described previously. As shown in Fig. 5, no change in functional performance was observed in either bioreactor. Microscopy observations suggest that the transition away from a *Plasticicumulans* dominated microbial community may have been faster in presence of chromium (Fig. S3, Supporting Information).

### Titration of additional iron

Iron measurements in filtered reactor effluent from ${\textit{SBR}_{{H_2}S{O_4}}}$ and ${\textit{SBR}_{HCl}}$ demonstrated that at least 50% of the influent iron (16 μM Fe) was detected in filtered reactor effluent samples. Even though considerable iron concentrations were found in both bioreactors, titration experiments analogue to the experiments with chromium and nickel were conducted with iron. The continuous addition of iron had a pronounced effect on the feast length as is displayed in Fig. 5. The functional response showed a remarkable similarity to the response observed when switching H_2_SO_4_ back to HCl Fig. 4.

## DISCUSSION

### Microbial competition is affected by the type of acid used for pH control

In this work we have demonstrated that in a pulse fed aerobic bioreactor fed with acetate as sole carbon and energy source, the type of acid (${\textit{SBR}_{{H_2}S{O_4}}}$ and ${\textit{SBR}_{HCl}}$) used as pH controlling agent has a paramount impact on the functional properties of the process and the microbial community established in steady state (Fig. 1). Switching the pH controlling agent resulted in the functional and microbial transition to the alternating state: ${\textit{SBR}_{{H_2}S{O_4} \to HCl}}$ became equivalent to ${\textit{SBR}_{HCl}}$ and vice versa. The reproducibility of these remarkable transitions was verified over twenty times with biomass from both ${\textit{SBR}_{HCl}}$ and ${\textit{SBR}_{{H_2}S{O_4}}}$. The changes in functional performance of the microbial communities throughout all transition experiments were highly comparable, which further emphasizes the dependency of the process on the acid used for pH control.

### Microbial competition is directly affected by iron leaching due to corroding reactor inlets

Additional experiments demonstrated that the type of anion supplied with the acid used for pH control only had a minor impact on the enrichment. Apparently, the differences observed were an indirect effect of the type of acid used for pH control. Abiotic experiments demonstrated that steel from the bioreactor corroded in HCl, and not in H_2_SO_4_ resulting in increased concentrations of iron, chromium and nickel (Fig. S2, Supporting Information). Independent titration experiments with these main constituents of 316L steel, demonstrated that supplementing ${\textit{SBR}_{{H_2}S{O_4}}}$ with chromium and nickel did not enable the establishment of a process equivalent to ${\textit{SBR}_{HCl}}$, nor the functional enrichment of *P. acidivorans* (Fig. 5). The presence of chromium was correlated with minor shifts in functionality and morphology and could therefore be a contributing factor to the microbial competition as observed in the cultivations (Lemire, Harrison and Turner 2013). The titration of iron did result in a steady state operational performance of ${\textit{SBR}_{{H_2}S{O_4}}}$ fully equivalent to ${\textit{SBR}_{HCl}}$ and enrichment of *P. acidivorans*. This led to the remarkable conclusion that some form of iron limitation was preventing the enrichment of *P. acidivorans* even though less than 50% of iron in the original medium was consumed.

### Iron bioavailability and its role in microbial competition

From the results in this research it was not apparent which factors affect the biological availability of iron for *P. acidivorans*. Iron is the most important micro nutrient for almost all microorganisms, and due to iron's complex speciation and precipitation properties microorganisms have evolved to scavenge iron at very low concentrations (Lankford and Byers 1973). Natural ecosystems show dissolved iron concentrations below 1 nM (Falkowski 1998). Neilands (1981) describes how microbes with high iron affinity due to siderophores and cognate transport apparatus still grow optimal at iron concentrations below 0.1 µM. The genome of *P. acidivorans* contains several high-affinity iron transporters (e.g.: catecholate siderophore receptor: PWV65540.1, iron ABC transporter: PWV65654.1, ferrous iron transporter: PWV63195.1), making it unlikely that the measured iron Fe(III) concentrations (>9 µM) are limiting its growth rate (Göker 2017).

Although iron was added to the medium as Fe(II), it oxidizes to Fe(III) in aerobic conditions, resulting in complex speciation (Davison and Seed 1983). EDTA was added as chelating agent to the medium to prevent precipitation of iron salts and oxides. The high binding strength of the EDTA-Fe(III) complex (k_f_Fe(III)_$ \approx $ 25) reduces the free iron concentration to the order of 10^−29^ M Fe(III) in the bioreactors, possibly affecting the iron uptake rate (Anderegg 1977). Additionally, the method of trace metal dosing was shown to influence microbial functionality in anaerobic digestion, where continuous titration at low concentrations achieved higher rates than pulse dosing excessive amounts (Gonzalez-Gil, Kleerebezem and Lettinga 1999). The oxidation state of iron, its complexation with chelating agents, and the manner in which iron is added to the medium, i.e. through pulse or titration, was demonstrated to play a key role in microbial competition and enrichment in this work.

### Transition state—altered functional performance of the microbial community.

${\textit{SBR}_{HCl}}$ enrichments dominated by *P. acidivorans* showed an almost instantaneous change in functional performance when the acid agent was switched to H_2_SO_4_ (Fig. 3). Despite the change in functional performance within 1 to 2 generations, the change in abundance of the dominant microbial community members seemed to take at least 10 generations as shown through 16S rRNA gene sequencing and microscopy (Fig. 2). The perceived change in function is related to microbial abundance and activity, here it is likely that the activity of the dominant community members changed. During the first cycles after the acid switch, the functional performance of *P. acidivorans* dominated enrichments showed high similarity to enrichments dominated by *Zoogloea sp*. The apparent decrease in the biomass specific substrate uptake rate upon the transition from HCl to H_2_SO_4_ as pH controlling agent diminishes the competitive advantage of *P. acidivorans* in the experiments described in this work, allowing the minor community members to increase in abundance during the consecutive cycles. Pure culture cultivations with *P. acidivorans* and *Zoogloea sp*. might aid in unraveling the specific mechanisms behind the current observation by looking at transcriptomics and proteomic changes under different iron limitations. These observations evidently raise the question to what extent we may overlook other limitations and corresponding differences in functional distinct behavior due to choices in bioreactor operation and medium composition.

### Secondary limitation may have an important impact on microbial community structure and functioning—The environment selects

From these experiments it becomes apparent that seemingly negligible operational differences may impact enrichment results. The results as observed in this study raise the question to which extent our assumption of single limiting factors in enrichment studies can be supported. Starting from the hypothesis of Baas Becking ‘everything is everywhere; but the environment selects’ (Baas-Becking 1934), the question arises how much diversity and alternative functionalities can be unlocked and are currently overlooked by the nuances of enrichment studies. Many microbiologists have anecdotal evidence where their tricks of the trade allowed them to cultivate and isolate specific microbial species. In most microbiology literature, minute differences often go unnoticed or undocumented, possibly hampering the elucidation of novel biological mechanisms. Some noteworthy exceptions include the works of Zeikus and Thauer in their respective labs on *Methanobacterium thermoautotrophicum* (Zeikus and Wolfe 1972). Eventually the difference in growth rate measured in both laboratories was related to the nickel in the needles in Zeikus’ lab (Schönheit, Moll, and Thauer 1979). And a more recent publication from the group of Op den Camp explains the growth dependencies of *Methylacidiphilum fumariolicum* on rare earth metals, which were present in medium supplemented with mudpot water from Solfatara (Pol *et al*. 2014).

The above-mentioned findings resulted from close observation and critical analysis of operational practices. In order to facilitate the discovery and understanding of secondary factors affecting enrichment studies, more systematic and comparative research is required.

## CONCLUSIONS

Aerobic, pulse fed sequencing batch bioreactors allow efficient enrichment of microbial communities with superior PHA storing capacity. This work has demonstrated that minute differences in medium composition may strongly affect microbial competition and therewith affect the PHA producing capacity significantly. Here we elucidated that the type of acid used for pH-control affected the bioavailability of iron and therewith determined the microbial community structure and the PHA producing capacity: Even though no iron limitation was observed in any of the systems, the titration of additional iron through corrosion of reactor inlets facilitated the enrichment of the well-known PHA producer *Plasticicumulans acidivorans*. By changing the hydrochloric acid for sulfuric acid as pH controlling agent, the corrosion of acid inlet points of the reactor halted and an immediate change in functional response was observed, followed by a change in microbial community towards a *Zoogloea sp*. dominated culture. The results described in this work demonstrate that apparently insignificant variations in medium composition can induce secondary nutrient limitations and have a major impact on the functional and structural development of microbial enrichments.

## Supplementary Material

fiab002_Supplemental_File
