# Supplementary material for: Seemingly trivial secondary factors may determine microbial competition: a cautionary tale on the impact of iron supplementation through corrosion
Source: FEMS Microbiol Ecol. 2021 Jan 11;97(2):fiab002. doi: 10.1093/femsec/fiab002 (PMC7878175; doi:10.1093/femsec/fiab002)
Supplement: fiab002_Supplemental_File [file fiab002_supplemental_file.docx]

**Supplementary Figure 1**: Feast length of $\boldsymbol{SBR}_{\boldsymbol{HCl\to}\boldsymbol{H}_{\boldsymbol{2}}\boldsymbol{SO}_{\boldsymbol{4}}}$ after switching the acid to H_2_SO_4_ (from cycle 10) during cultivation with increased NaCl (from cycle 20), 20mM in light triangles and 40mM in dark triangles. *P. acidivorans* remained the most abundant species in ${SBR}_{HCl\to H_{2}{SO}_{4}}$ for 30 cycles with the NaCl supplemented medium, but eventually the community shifted towards a community dominated by *Zoogloea sp.* after 40 cycles (**Supplementary Figure 6**).

Supplementary Table 1: The composition of type 316L stainless steel from which the reactor inlet is made

| Compound | 316L steel |
| --- | --- |
|  | % ^a^ |
| Carbon | 0.03 max. |
| Sulfur | 0.03 max. |
| Phosphorus | 0.045 max. |
| Nitrogen | 0.1 max. |
| Silicon ^b^ | 0.75 max. |
| Manganese | 2.00 max. |
| Molybdenum | 2.00 - 3.00 |
| **Chromium ^b^** | **16.00 - 18.00** |
| **Nickel ^b^** | **10.00-14.00** |
| **Iron** | **Balance** |
| ^a^ Weight percentage (g/g)  ^b^ Element not present in media (Supplementary Table 2) | |


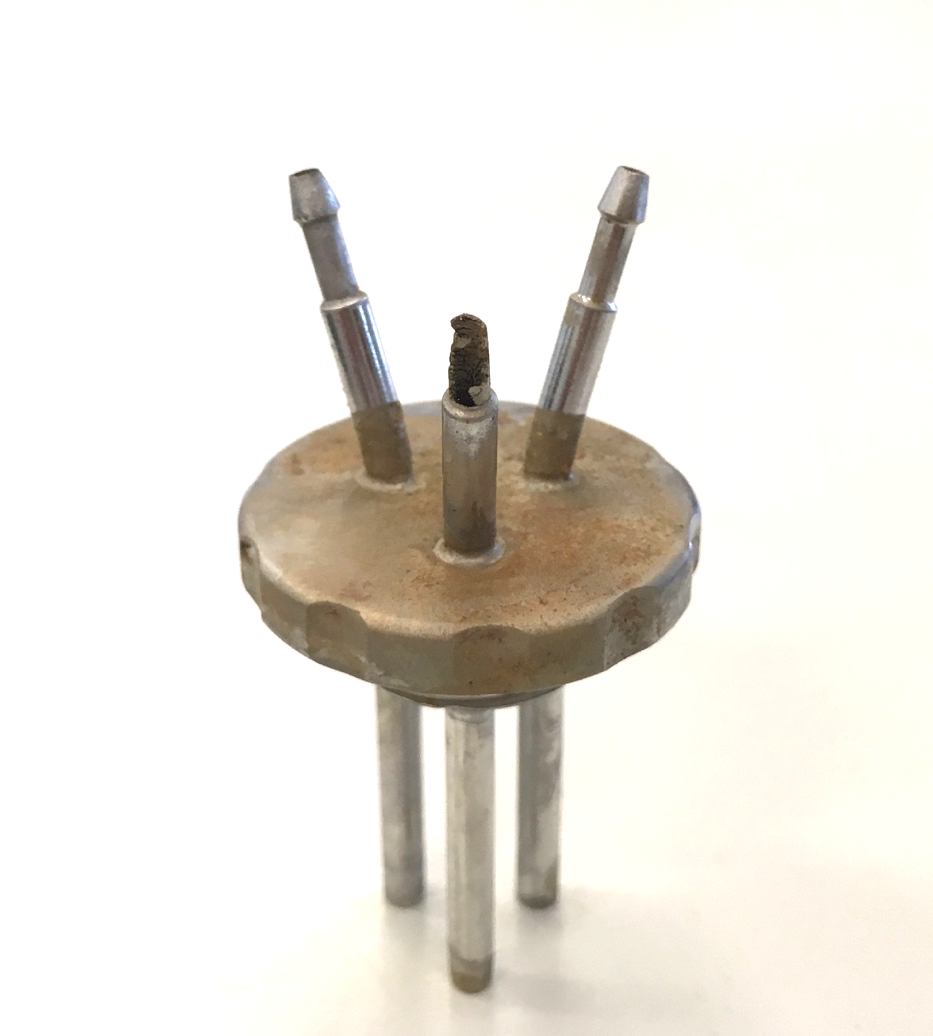

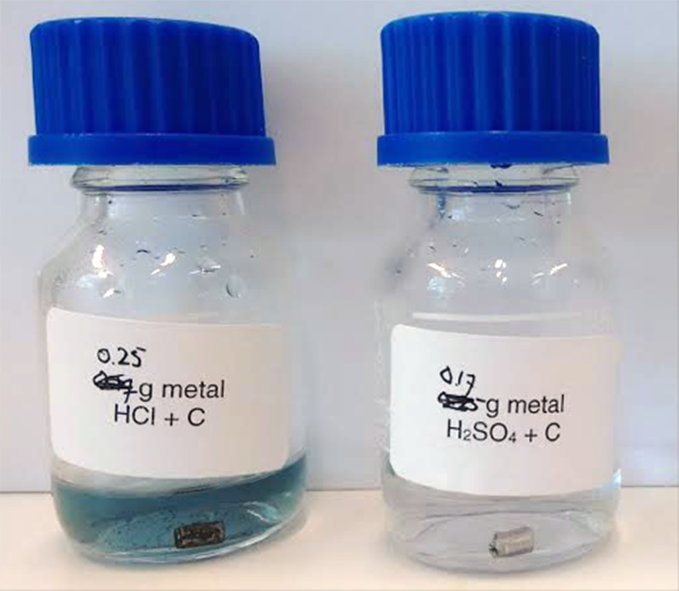


Supplementary Figure 2: Photographs of an inlet triplet (left) of which the acid inlet is significantly corroded, and two acid bottles (right) with 5 mL 1 M HCl, and 5 mL 0.5 M H_2_SO_4_ with a small piece of 316L reactor metal after 30 days.

Supplementary Table 2: Composition of the trace elements solution, and relative contribution of leaching on reactor medium.

| Compound | MW | Concentration | | Reactor feed | Inlet leaching ^b^ |
| --- | --- | --- | --- | --- | --- |
|  | g/mol | (g/L) | (mmol/L) | $\mu mol/L$ | $\mu mol/L$ |
| EDTA Titriplex III ^a^ | 372 | 63.69 | 171 | 256 |  |
| ZnSO4.7H2O | 288 | 22.00 | 77 | 116 |  |
| CaCl2.H2O | 147 | 7.34 | 50 | 75 |  |
| MnCl2.4H2O | 198 | 5.06 | 26 | 39 |  |
| FeSO4.7H2O | 278 | 2.99 | 11 | 16.5 | 35 - 140 |
| CoCl2.6H2O | 238 | 1.61 | 7 | 10.5 |  |
| CuSO4.5H2O | 250 | 1.51 | 6 | 9 |  |
| (NH4)6Mo7O24.4H2O | 1164 | 1.10 | 1 | 1.5 |  |
| NiSO4.H2O |  |  |  | - | 7 - 30 |
| CrK(SO4)2.H2O |  |  |  | - | 10- 40 |
| ^a^ EDTA chelator is added in equal molar ratio to trace metals  ^b^ Estimation of the inlet concentration due to leaching as described below in Supplementary Table 3 | | | | | |


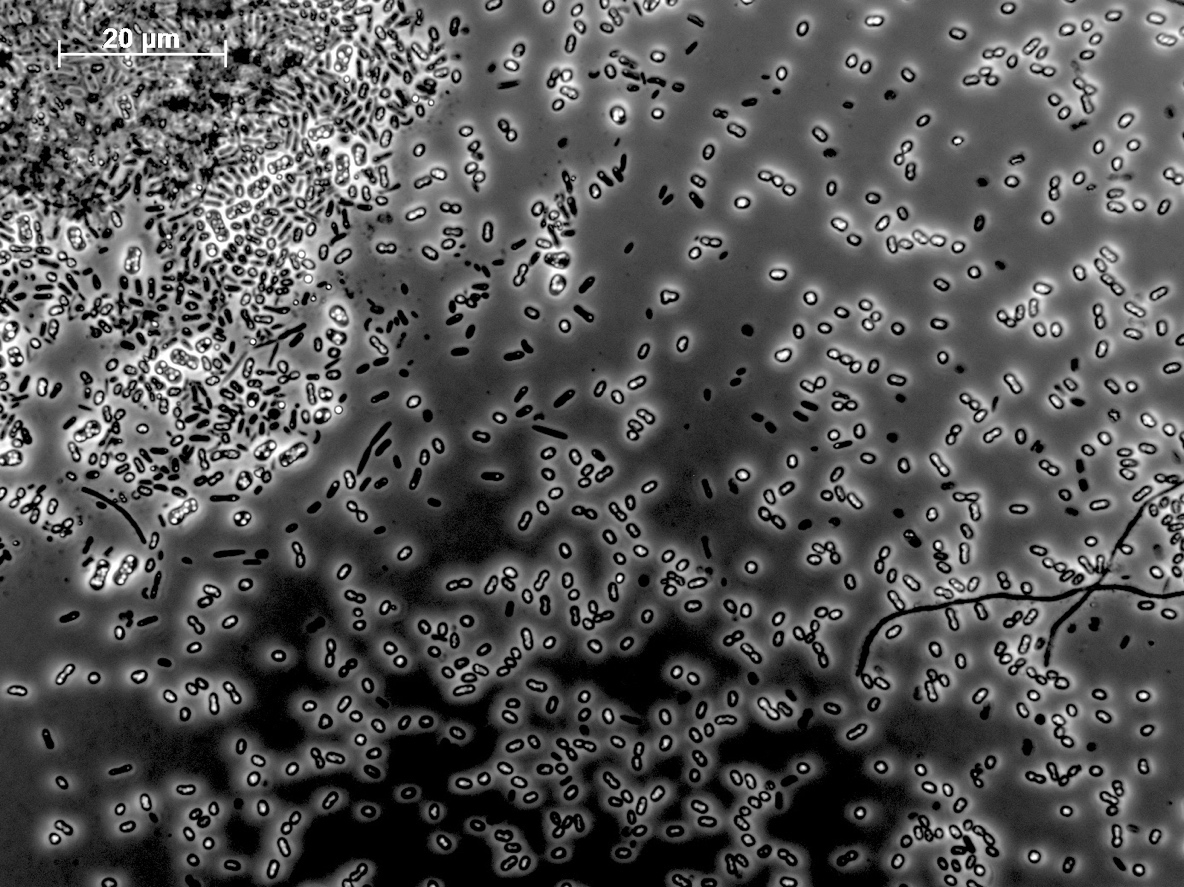

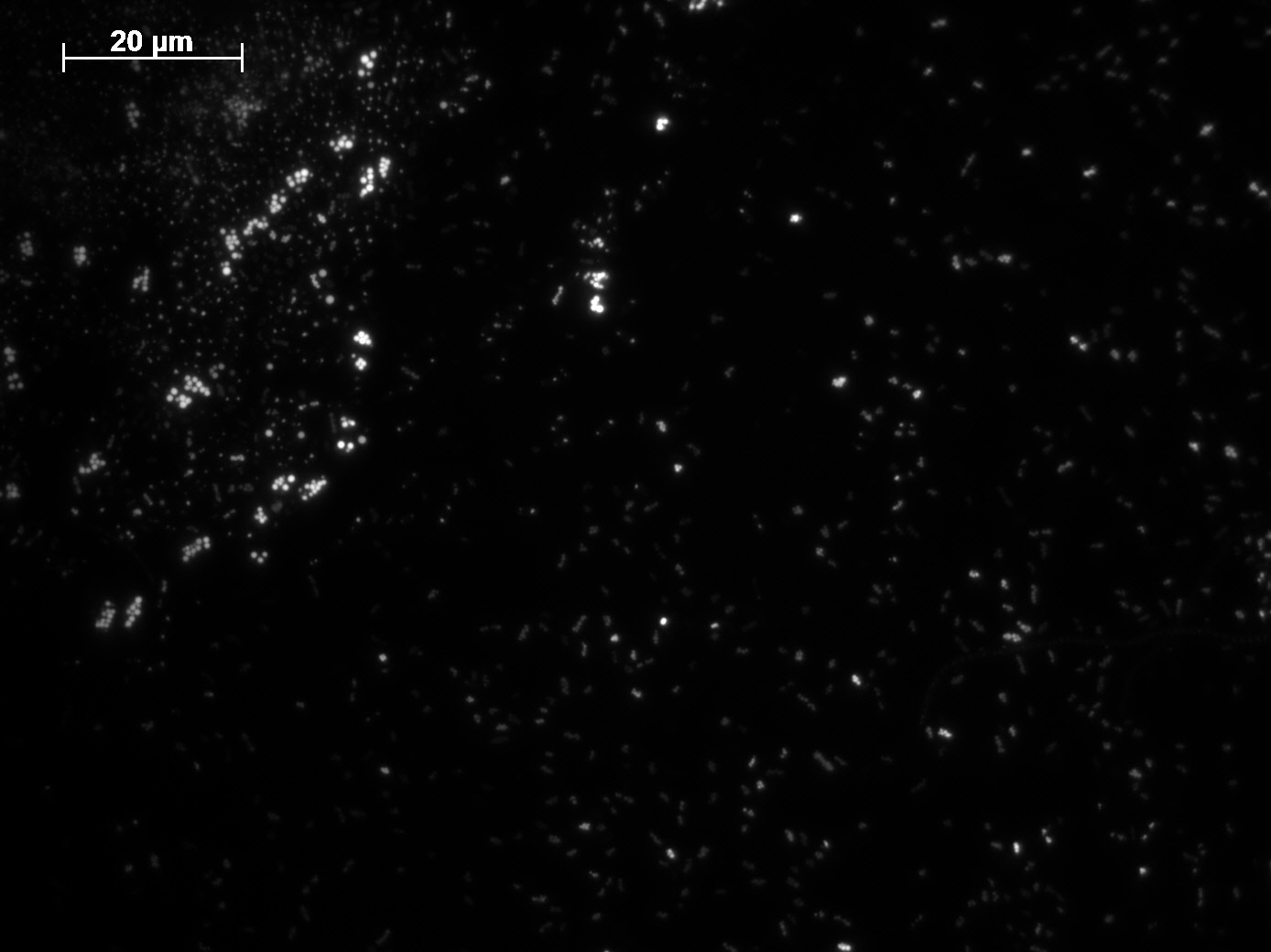


Supplementary Figure 3: Microscopic images of the microbial culture in $\boldsymbol{SBR}_{\boldsymbol{HCl\to}\boldsymbol{H}_{\boldsymbol{2}}\boldsymbol{SO}_{\boldsymbol{4}}}$ with additional chromium at the end of the feast phase in dark field (top) and BODIPY 505/515 PHA staining (bottom). This image is taken after ten operational cycles at a chromium concentration of approximately 20μM in the bioreactor. The morphology of the microbial cells is distinct from those in stable HCl and H_2_SO_4_ enrichments, and from biomass in the transition state.

The presence of chromium is an additional factor that could contribute to microbial competition as observed in the cultivations (Lemire, Harrison, and Turner 2013).

Supplementary Figure 4: Visual representation of difference in PHA production capacity when expressed as dry weight fraction. For each gram of catalytic biomass that is produced (blue), *n* grams of PHA need to be produced (orange) to reach the PHA storage capacity as described on the x-axis.

Supplementary Figure 5: Relative abundance of microbial community structure based on 16S rRNA-gene Amplicon sequencing for bioreactor $\boldsymbol{SB}\boldsymbol{R}_{\boldsymbol{HCl}}$ at four timepoints. SBR-HCl indicates the community composition before a shift in acid for pH control to H_2_SO_4_, and 10, 20, and 34 cycles after the shift.

During the transition from a *P. acidivorans* dominated culture to *Zoogloea sp.* no specific transient micro-organisms are detected with 16S rRNA-gene Amplicon sequencing. The running theory proposed in this manuscript is that some form of iron limitation occurs during cultivation with H_2_SO_4_, which suggests that increased abundance is expected of microbes with a higher iron affinity, or iron uptake mechanism. Secondly, species with a decreased iron affinity that are initially present (e.g: *P. acidivorans*) could suffer from decreased viability and decay. This would also allow microbes that grow on complex substrates to transiently proliferate. This mechanism if elaborated by Stouten and colleagues (2019).

Supplementary Figure 6: Relative abundance of microbial community structure based on 16S rRNA-gene Amplicon sequencing for bioreactor $\boldsymbol{SB}\boldsymbol{R}_{\boldsymbol{H}_{\boldsymbol{2}}\boldsymbol{S}\boldsymbol{O}_{\boldsymbol{4}}}$ at four timepoints. SBR-H_2_SO_4_ indicates the community composition before a shift in acid for pH control to HCl, and 10, 20, and 34 cycles after the shift.

Contrary to the shift describe above (**Supplementary Figure 5**), during the transition from a *Zoogloea sp.* dominated culture to *P. acidivorans* specific transient micro-organisms are detected with 16S rRNA-gene Amplicon sequencing. In this system the bioavailability of iron likely increases, allowing a transient high abundance of the *Flavobacterium*. Its abundance aligns with an increase in the feast length (**Figure 3** of manuscript), signifying that its competitiveness might not be related to carbon substrate uptake rate. Possible competitive factors include opportunistic growth on decaying cell matter by hydrolysis of complex substrates (Bernardet 2002), and alternative complex III (ACIII) used in the respiration chain which is more iron dependent (Sun et al. 2018). The transient nature could be explained by the superior carbon substrate uptake rates of *P.* *acidivorans*, and its maintained cell viability (Stouten et al. 2019).

Supplementary Figure 7: Relative abundance of microbial community structure based on 16S rRNA-gene Amplicon sequencing for bioreactor $\boldsymbol{SB}\boldsymbol{R}_{\boldsymbol{HCl\to}\boldsymbol{H}_{\boldsymbol{2}}\boldsymbol{S}\boldsymbol{O}_{\boldsymbol{4}}}$ at two timepoints, 30 and 40 cycles after the start of the titration of NaCl to $\boldsymbol{SB}\boldsymbol{R}_{\boldsymbol{HCl\to}\boldsymbol{H}_{\boldsymbol{2}}\boldsymbol{S}\boldsymbol{O}_{\boldsymbol{4}}}$.

Supplementary Table 3: Calculation of corrosion and titration of acid inlet feed triplet.

| **Calculations leaching experiment HCl** | | | 30 | **days in acid** | |  | |  |
| --- | --- | --- | --- | --- | --- | --- | --- | --- |
| Mass: | 250 | mg |  |  | |  | |  |
| Volume HCl | 5 | ml |  |  | |  | |  |
| Measurement dilution | 100 | x |  |  | |  | |  |
| Measured concentration | 2.54 | mg Fe/L |  |  | |  | |  |
|  |  |  |  |  | |  | |  |
| **Concentrations based on measurement and steel composition:** | | | | ~ Composition 316L | | | | MW |
| Fe | 254 | mg Fe/L |  | Fe | 62% | | 56 | |
| Cr | 74 | mg Cr/L |  | Cr | 18% | | 52 | |
| Ni | 57 | mg Ni/L |  | Ni | 14% | | 59 | |
| Mo | 8 | mg Mo/L |  | Mo | 2% | | 96 | |
| Mn | 8 | mg Mn/L |  | Mn | 2% | | 55 | |
|  |  |  |  |  | |  | |  |
| Total metal leached | 2.048 | mg |  |  | |  | |  |
|  | 0.82% | corroded in 30 days | |  | |  | |  |
|  |  |  |  |  | |  | |  |
| **Estimation of number of days until leakage at continuous exposure to (fresh) acid** | | | | | | | |  |
| Breakpoint occurs when | 10% | of pipe has corroded (Assumption) | | | |  | |  |
|  | 366 | days until breakpoint | |  | |  | |  |
|  |  |  |  |  | |  | |  |
| **Estimation of additional supplemental iron due to leaching** | | | |  | |  | |  |
| Dosed | 27 | ml acid / cycle | |  | |  | |  |
| Concentration Fe in acid | 51 | mg Fe / L |  | (low estimate) | | | |  |
|  | 254 | mg Fe / L |  | (high estimate) | | | |  |
| Dosed iron per cycle | 0.025 | mmol Fe / cycle | | (low estimate) | | | |  |
|  | 0.123 | mmol Fe / cycle | | (high estimate) | | | |  |
|  |  |  |  |  | |  | |  |
| **Stock solutions (derived from leaching experiment)** | | | |  | |  | |  |
| Iron | 5.0 | mM Fe |  |  | |  | |  |
| Chromium | 1.5 | mM Cr |  |  | |  | |  |
| Nickel | 1.0 | mM Ni |  |  | |  | |  |
|  |  |  |  |  | |  | |  |
| **Titration experiment ranging from low to high estimate** | | | |  | |  | |  |
| Flow rate titration | 5 | ml/12 hour |  | (low estimate) | | | |  |
|  | 20 | ml/12 hour |  | (high estimate) | | | |  |
|  |  |  |  |  | |  | |  |
| **Supplemented metals** | low | high |  |  | |  | |  |
|  | 0.025 | 0.100 | mmol Fe / cycle | | |  | |  |
|  | 0.008 | 0.030 | mmol Cr / cycle | | |  | |  |
|  | 0.005 | 0.020 | mmol Ni / cycle | | |  | |  |

**Supplementary References**

Bernardet, J. F. 2002. “Proposed Minimal Standards for Describing New Taxa of the Family Flavobacteriaceae and Emended Description of the Family.” *International Journal of Systematic and Evolutionary Microbiology* 52 (3): 1049–70. https://doi.org/10.1099/ijs.0.02136-0.

Lemire, Joseph A., Joe J. Harrison, and Raymond J. Turner. 2013. “Antimicrobial Activity of Metals: Mechanisms, Molecular Targets and Applications.” *Nature Reviews Microbiology* 11 (6): 371–84. https://doi.org/10.1038/nrmicro3028.

Stouten, Gerben Roelandt, Carmen Hogendoorn, Sieze Douwenga, Estelle Silvia Kilias, Gerard Muyzer, and Robbert Kleerebezem. 2019. “Temperature as Competitive Strategy Determining Factor in Pulse-Fed Aerobic Bioreactors.” *The ISME Journal*, September. https://doi.org/10.1038/s41396-019-0495-8.

Sun, C., S. Benlekbir, P. Venkatakrishnan, Y. Wang, S. Hong, J. Hosler, E. Tajkhorshid, J.L. Rubinstein, and R.B. Gennis. 2018. “Structure of the Alternative Complex III in a Supercomplex with Cytochrome Oxidase.” *Nature* 557 (7703): 123–26. https://doi.org/10.1038/s41586-018-0061-y.
